# Does sampling using random digit dialling really cost more than sampling from telephone directories: Debunking the myths

**DOI:** 10.1186/1471-2288-6-6

**Published:** 2006-02-24

**Authors:** Baohui Yang, Margo Eyeson-Annan

**Affiliations:** 1 Centre for Epidemiology and Research, Population Health Division, New South Wales Department of Health, Locked Bag 961, North Sydney, New South Wales, 2059, Australia

## Abstract

**Background:**

Computer assisted telephone interviewing (CATI) is widely used for health surveys. The advantages of CATI over face-to-face interviewing are timeliness and cost reduction to achieve the same sample size and geographical coverage. Two major CATI sampling procedures are used: sampling directly from the electronic white pages (EWP) telephone directory and list assisted random digit dialling (LA-RDD) sampling. EWP sampling covers telephone numbers of households listed in the printed white pages. LA-RDD sampling has a better coverage of households than EWP sampling but is considered to be more expensive due to interviewers dialling more out-of-scope numbers.

**Methods:**

This study compared an EWP sample and a LA-RDD sample from the New South Wales Population Health Survey in 2003 on demographic profiles, health estimates, coefficients of variation in weights, design effects on estimates, and cost effectiveness, on the basis of achieving the same level of precision of estimates.

**Results:**

The LA-RDD sample better represented the population than the EWP sample, with a coefficient of variation of weights of 1.03 for LA-RDD compared with 1.21 for EWP, and average design effects of 2.00 for LA-RDD compared with 2.38 for EWP. Also, a LA-RDD sample can save up to 14.2% in cost compared to an EWP sample to achieve the same precision for health estimates.

**Conclusion:**

A LA-RDD sample better represents the population, which potentially leads to reduced bias in health estimates, and rather than costing more than EWP actually costs less.

## Background

Computer assisted telephone interviewing (CATI) is widely used for health surveillance surveys. The main advantages of CATI over the traditional face-to-face interviewing are timeliness and substantial cost reduction to achieve the same sample size and geographical coverage via residential telephones. In Australia, there is little bias in population-based health estimates due to a high residential telephone coverage of 97.5%, [1] combined with post stratification weighting to the population.

Even though full coverage of household telephones can be achieved by simple random digit dialling (RDD), it is not efficient due to inclusion of too many numbers that are not currently household telephone numbers (out-of-scope). The Waksberg Method improves the efficiency of RDD by using a two-stage sampling procedure, [2,3] and this method is widely used in studies outside Australia [4]. When relatively good quality lists of household telephones are available, and the list itself does not have satisfactory coverage of the target population, information in the list (such as prefixes) can be used to develop a sampling frame that has a better coverage of households: this is called list assisted random digital dialling (LA-RDD). LA-RDD further improves the efficiency of simple RDD by removing banks of numbers with no listed household telephone numbers.

Two major CATI sampling procedures are used in health surveys: sampling directly from the electronic white pages (EWP) telephone directory, [5-7] and LA-RDD sampling [7,8]. The EWP covers telephone numbers of households who choose to have their telephone listed in the printed white pages telephone directory. The proportion of households who choose to not have their telephone listed in the white pages telephone directory (that is, unlisted or silent numbers) is estimated to be at least 15% in Australia, on the basis of a comparison of the estimated number of households with telephones and the number of listings in the EWP [9].

The LA-RDD sampling surveys have a better coverage of households than sampling directly from the EWP but are considered to be more expensive due to interviewers dialling more out-of-scope numbers. Bennett and Steel estimate that, compared with sampling directly from the EWP, using lists of active telephone numbers for each telephone exchange increases total interviewer time by about 20%, and if LA-RDD were used the increase in interviewing time could be about 12% [9]. Wilson and Starr argue that the EWP is preferred due to low cost based on number of calls to get an interview (1.6 calls using the EWP sampling methodology compared with 6.5 calls using the Waksberg RDD sampling methodology) [10].

Results of studies examining the effects of bias due to sample methodology (EWP versus RDD) on health estimates for the general population have varied. For example, Bennett and Steel found that EWP sampling led to significant bias in unweighted estimates for households who had moved, single parent households, and households composed of unrelated people [9]. Wilson and Starr found some difference, however, in demographic profiles between RDD and EWP sampling methodologies, but minimal bias was found in the studied health estimates from the EWP sample compared with a RDD sample after post-stratification weighting [10].

In addition to the limitations of no post stratification weighting in Bennett and Steel, and using the Waksberg RDD sampling methodology in Wilson and Starr, neither study used survey-quality and design-efficiency measures. These survey-quality and design-efficiency measures include:

• coefficients of variation in weights, where a larger coefficient of variation in weights means more extreme adjustment is needed for the sample to match the population benchmark;

• design effects, which are ratios of variance of estimates of a sampling method compared with a simple random sampling design.

In both Bennett and Steel and Wilson and Starr, the comparative costs of RDD and EWP sample surveys were based on achieving the same sample size, which is misleading when the design effects were different between the two survey designs.

The aims of this study are to use the 2003 data from the New South Wales Population Health Survey,[7] a CATI health survey in New South Wales, Australia, to:

• examine the demographic profiles of weighted and unweighted LA-RDD and EWP samples;

• compare a series of health estimates from the LA-RDD and EWP samples;

• compare the costs of LA-RDD and EWP on the basis of achieving the same precision of estimates, rather than on the basis of achieving the same sample sizes.

## Methods

### Sample generation

A stratified sample was generated using the LA-RDD method for the New South Wales Population Health Survey in 2003. The LA-RDD sampling frame was created by using four digit prefixes that contained connected residential numbers from the EWP. All possible suffixes were then added to create a list of possible telephone numbers. This list was checked against the EWP so that banks of 10 continuous numbers with no numbers in the EWP were removed from the sampling frame. The list was further matched with an electronic business telephone directory to remove business numbers and fax numbers, to improve the efficiency of the final LA-RDD sampling frame. Each selected household telephone number was flagged as either EWP listed or unlisted (silent). So the resulting LA-RDD sampling frame includes all the EWP listed household telephone numbers and other potentially high productive telephone numbers with the four digit prefixes appearing in the EWP.

### Interviewing methodology

Letters of approach introducing the survey were sent to selected households with EWP listed telephones. Using a CATI facility, up to seven calls were made to establish initial contact with a household, and up to five more calls were made in order to contact a selected respondent and conduct an interview. Within a selected household, one person was selected at random from the usual residents in the selected household. Children were included in the survey, with information provided by a person aged over 16 years.

### Sample and weighting

The total number of completed interviews for the 2003 survey is 15,837, among which 12,403 had EWP listed household telephones. Of these, 13,008 interviews were adults (aged 16 years or over), among which 10,386 had EWP listed household telephones. The same method of weighting was applied to the whole LA-RDD sample and the EWP sample. Weighting for samples was by probability of selection, then post-stratification weighting was applied to adjust the sample distribution to match the Australian Bureau of Statistics 2003 mid-year residential population estimates jointly by gender, five-year age groups (0–4 years to 80 years and over), and geographical regions [11].

After weighting, other health related demographics, such as country of birth, speaking a language other than English at home, having indigenous origin, marital status, and quartiles of socioeconomic index for areas, were compared to the New South Wales population using the Australian Bureau or Statistics Census 2001 figures.

### Telephone call outcomes

Operational data for the survey were downloaded using SAWTOOTH WinCati version 4.1. The data included the following information for each attempted 'telephone' number, including connected and non-connected numbers: the number dialled; the number of attempts of dialling to that number; the starting and ending time for each dialling attempt to the number; whether or not the number is listed in the EWP; and whether the number dialled has led to a completed interview, or no answer, or a refusal, or a non-connected number, or any kind of out-of scope number (including non-connected numbers, fax machines, unusual tones, business-institution numbers, and households not eligible). Diallings to a non-connected number, or a number with no answer, or a number with unusual tone, were treated as non-costed diallings. For each attempted number, the sum of calling time over all attempts, the total number of attempted diallings, and the total number of costed diallings were calculated. The total costed diallings was zero for a non-connected number, or a number with no answer, or a number with unusual tone. Then the total calling time, total number of diallings, total number of costed diallings, and total number of completed interviews, were calculated over all the attempted numbers to obtain the whole LA-RDD sample and over all the EWP listed numbers to obtain the EWP sample respectively. Finally, the average calling time, average number of diallings, and average number of costed diallings, were calculated per LA-RDD completed interview and per EWP completed interview.

Response rates were calculated as the number of completed interviews divided by the sum of the number of completed interviews and number of refusals.

### Estimate comparison

Prevalence estimates were generated using PROC SURVEYMEANS in SAS version 8.2. Pairs of estimates on 23 health-related indicators for males, females, and overall persons aged 16 years and over were calculated from a weighted EWP sample and a weighted LA-RDD sample. Differences between the point estimates were examined. Detailed information on the definition of these indicators can be found in the electronic report for the New South Wales Population Health Survey in 2003 [12].

### Coefficient of variation and design effects

Coefficients of variation in weight were produced by PROC SURVEYMEANS in SAS version 8.2 from the EWP sample and LA-RDD sample respectively. The design effect for each indicator was calculated by dividing the variance of the prevalence estimate (the square of the standard error obtained from PROC SURVEYMEANS in SAS) by the estimated variance of the corresponding estimate from an assumed simple random sample, with the same sample size, *Var*_*srs *_= p^
 MathType@MTEF@5@5@+=feaafiart1ev1aaatCvAUfKttLearuWrP9MDH5MBPbIqV92AaeXatLxBI9gBaebbnrfifHhDYfgasaacH8akY=wiFfYdH8Gipec8Eeeu0xXdbba9frFj0=OqFfea0dXdd9vqai=hGuQ8kuc9pgc9s8qqaq=dirpe0xb9q8qiLsFr0=vr0=vr0dc8meaabaqaciaacaGaaeqabaqabeGadaaakeaacuWGWbaCgaqcaaaa@2E25@(1 - p^
 MathType@MTEF@5@5@+=feaafiart1ev1aaatCvAUfKttLearuWrP9MDH5MBPbIqV92AaeXatLxBI9gBaebbnrfifHhDYfgasaacH8akY=wiFfYdH8Gipec8Eeeu0xXdbba9frFj0=OqFfea0dXdd9vqai=hGuQ8kuc9pgc9s8qqaq=dirpe0xb9q8qiLsFr0=vr0=vr0dc8meaabaqaciaacaGaaeqabaqabeGadaaakeaacuWGWbaCgaqcaaaa@2E25@)/*n*. Coefficient of variation in weights and overall design effects for individual indicators and average design effect across these indicators were compared between the two samples.

### Cost comparison

Comparative cost data for the EWP sample and the LA-RDD sample were extracted, including number of dialling, number of costed dialling, and length of calling time. The EWP sample and LA-RDD sample costs were compared on the basis of achieving the same precision of estimates as that for an assumed simple random sample of 1,000 respondents, taking into consideration the different design effects of the EWP sample and RDD sample. Estimated costs are in Australian dollars using the average charge per telephone call of A$0.20, and the calling cost of A$20.00 an hour for a CATI interviewer, with the overall cost calculated as:

*Cost = A$0.20 × number of costed dialling + A$20 × calling time in hours*.

## Results

### Quality of sampling frames and response rates

Among all the numbers dialled to get the 15,837 completed interviews, 48% were out of scope, which included fax machine numbers, not connected numbers, unusual tone numbers, business or institution numbers, and household numbers that were not in New South Wales. Among the EWP listed numbers dialled, 26% were out of scope numbers.

Among the 15,837 completed interviews, 3,434 (21.7%) were from households with non-EWP-listed telephone numbers. Of the 13,008 interviews on adults (aged 16 years or over), 2,622 (20.2%) were from households with non-EWP-listed telephone numbers.

The response rate for the EWP sample was 71.4% and 69.0% for the overall LA-RDD sample.

#### Distribution of age group and gender of unweighted samples

**Figure 1:**
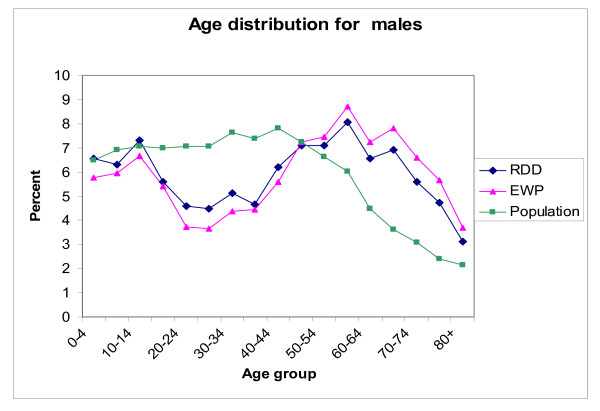
Age distribution of unweighted electronic white pages and list assisted random digit dialling survey samples in the New South Wales Population Health Survey in 2003 versus the New South Wales population: Males Source: New South Wales Health Survey Program, Centre for Epidemiology and Research, New South Wales Department of Health.

**Figure 2:**
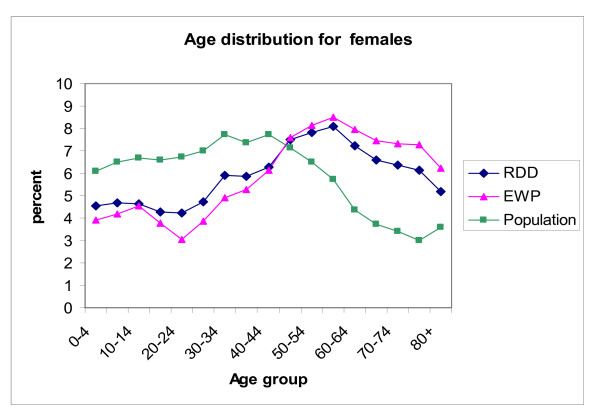
Age distribution of unweighted electronic white pages and list assisted random digit dialling survey samples in the New South Wales Population Health Survey in 2003 versus the New South Wales population: Females. Source: New South Wales Health Survey Program, Centre for Epidemiology and Research, New South Wales Department of Health.

The proportions for males in both the unweighted LA-RDD sample (43.1%) and the EWP sample (42.7%) were under-represented compared to the overall New South Wales population (49.7%). In both males and females the LA-RDD sample was a closer fit to the population five-year age group distribution than the EWP sample (Figures 1 and Figure 2).

The coefficients of variation in weights are estimated as 0.96 and 1.13 for the LA-RDD sample and EWP sample respectively. The coefficient of variation in weights are 1.03 and 1.21 for the LA-RDD adult sample and EWP adult sample respectively. As shown, a larger coefficient of variation of weights for the EWP sample indicates that more adjustment is needed for the EWP sample to match the population benchmark than for the LA-RDD sample.

### Demographics of weighted samples

Both the LA-RDD and the EWP weighted samples over-estimate people who were born in Australia, indigenous Australians, and married people, and under-estimated people who speak a language other than English at home (Table 1). For each of the demographics, however, the weighted LA-RDD sample was closer to the New South Wales population [13]. The weighted LA-RDD sample is also slightly closer to the New South Wales population in the proportion of socioeconomic index quartiles [14].

**Table 1:** Demographics of weighted list assisted random digit dialling sample and weighted electronic white pages sample from the New South Wales Population Health Survey in 2003 compared with demographics of the New South Wales population (Australian Bureau of Statistics 2001 Census)

**Demographic**	**LA-RDD %**	**EWP %**	**New South Wales %**	
			2001 Census*	2003 Estimate**

Born in Australia	78.4	79.9	70.5	69.5
				
**Non-English speaking at home**	14.0	12.4	24.3	24.5
				
Indigenous	2.07	1.85	1.9	2.0
				
Marital status				
				
Married (registered)	60.97	62.92	51.7	50.5
Widowed	4.66	4.64	6.5	6.6
Separated but not divorced	3.14	2.53	3.3	3.5
Divorced	6.36	5.67	7.2	7.5
Never married	24.86	24.24	31.3	31.5

**Social Economic Index for Areas**				
				
First quartile	20.33	19.71	25	25
Second quartile	25.96	25.91	25	25
Third quartile	23.75	23.64	25	25
Fourth quartile	29.96	30.75	25	25

* ABS 2001 Census. [13]

** Estimate projected from the 1991, 1996 and 2001 ABS Census. [15]

Source: New South Wales Health Survey Program, Centre for Epidemiology and Research, New South Wales Department of Health.

### Comparison on estimates for health related indicators for adults

The 23 health indicators examined included health behaviours, health status, health services, and health related social capital for adults aged 16 years and over. The weighted prevalence estimates for these health indicators from the LA-RDD sample and the EWP sample are listed in Table 2 for males, females, and persons respectively.

**Table 2:** Health estimates for weighted list assisted random digit dialling sample and weighted electronic white pages sample in the New South Wales Population Health Survey in 2003

Indicators	**Males**			**Females**			**Persons**		
			
	**LA-RDD %**	**EWP %**	**Diff %**	**LA-RDD %**	**EWP %**	**Diff %**	**LA-RDD %**	**EWP %**	**Diff %**
**Health Behaviour**									
Any alcohol risk drinking	41.1	42.3	1.2	30.1	29.9	-0.2	35.5	36.0	0.5
High alcohol risk drinking	17.7	18.1	0.4	10.8	9.7	-1.1	14.5	14.2	-0.3
Hand washing when handling raw meat	56.4	55.2	-1.2	64.3	63.3	-1	60.7	59.7	-1
Food insecurity in the last 12 months	5.3	4.8	-0.5	6.8	5.6	-1.2	6.0	5.2	-0.8
Recommended daily fruit intake	39.3	39.9	0.6	52.7	53.0	0.3	46.1	46.5	0.4
Usual use of low fat, reduced fat or skim milk	37.2	39.0	1.8	51.0	51.6	0.6	44.2	45.4	1.2
Adequate physical activity	49.3	50.0	0.7	40.2	40.2	0	44.7	45.0	0.3
Use of public water as usual source of water	81.6	81.5	-0.1	80.9	81.8	0.9	81.2	81.7	0.5
Current daily or occasional smoking	24.8	24.0	-0.8	19.7	18.2	-1.5	22.2	21.0	-1.2
Smoke free household	82.1	83.0	0.9	83.0	83.1	0.1	82.6	83.1	0.5
Recommended daily vegetable intake	11.9	11.7	-0.2	26.8	26.3	-0.5	19.5	19.1	-0.4
**Health Status**									
Ever diagnosed with asthma	19.3	18.9	-0.4	22.6	22.0	-0.6	21.0	20.5	-0.5
Current asthma	9.1	9.0	-0.1	12.6	12.0	-0.6	10.9	10.5	-0.4
Overweight or obesity	55.8	55.1	-0.7	41.2	40.9	-0.3	48.5	48.0	-0.5
Diabetes or high blood sugar	7.1	6.7	-0.4	5.7	5.5	-0.2	6.4	6.1	-0.3
Self-rated health status as excellent, very good or good	81.6	81.6	0	79.6	79.8	0.2	80.6	80.7	0.1
High or very high psychological distress	9.3	8.8	-0.5	12.8	12.8	0	11.1	10.9	-0.2
**Health Service**									
Emergency department care rated as excellent, very good or good.	80.3	81.4	1.1	77.9	77.3	-0.6	79.1	79.4	0.3
Difficulty getting health care when needing it	11.4	11.1	-0.3	15.0	14.6	-0.4	13.2	12.9	-0.3
Over night hospital care in the previous 12 months rated as excellent, very good or good	93.1	93.3	0.2	89.8	90.3	0.5	91.3	91.7	0.4
**Health Related Social Capital**									
Attended at least one community event in the previous six months	53.9	54.9	1	61.7	61.5	-0.2	57.9	58.2	0.3
Trust most people	71.6	72.8	1.2	68.0	69.2	1.2	69.8	71.0	1.2
Visit neighbours in the past week	67.0	68.0	1	63.8	64.7	0.9	65.3	66.3	1

Source: New South Wales Health Survey Program, Centre for Epidemiology and Research, New South Wales Department of Health.

Overall, for the adult population, the weighted prevalence estimates from the EWP sample compared to the LA-RDD sample was higher by:

• 1.0% or more for three indicators: usual use of low or reduced fat or skim milk, trust most people, and visit neighbours;

• 0.5–0.9% for three indicators: any alcohol risk drinking, use of public water as usual source of water, and smoke-free household;

• less than 0.5% for six indicators: self-rated health, positive emergency department rating, positive hospital care rating, attended at least one community event in the previous six months, recommended daily fruit intake, and adequate physical activity.

Overall, for the adult population, the weighted prevalence estimates from the EWP sample compared to the LA-RDD sample was lower by:

• 1.0% or more for two indicators: hand washing when handling raw meat, and current daily or occasional smoking;

• 0.5–0.9% for three indicators: ever diagnosed with asthma, overweight or obesity, and food insecurity in the last 12 months;

• less than 0.5% for six indicators: high alcohol risk drinking, diabetes or high blood sugar, high or very high psychological distress, difficulties in getting health care when needing it, recommended daily vegetable intake, and current asthma.

### Survey efficiency

On average, the design effect is 2.00 for the LA-RDD sample and 2.38 for the EWP sample across the 23 indicators studied (Table 3). Both the mean and median design effect ratios (design effect of EWP sample divided by design effect of LA-RDD sample) is 1.19. Therefore, on average, 19% more of the LA-RDD sample size is needed for the EWP sample to achieve the same precision for the health estimates. The largest five design effect ratios are for overnight hospital care rated as excellent, very good or good (1.35); high or very high psychological distress (1.32); current daily or occasional smoking (1.25); any high alcohol risk drinking (1.23); and trust most people (1.23). This means that, to achieve the same precision of the estimates on these indicators, 23% to 35% more of the LA-RDD sample size is needed for EWP sample.

**Table 3:** Design effects and design effect ratios of list assisted random digit dialling sample and electronic white pages sample in the New South Wales Population Health Survey in 2003 on 23 health related indicators for adults aged 16 years or older.

**Indicators**	**LA-RDD Design effect**	**EWP Design effect**	**Ratio of design effect**
**Health Behaviour**			
Any alcohol risk drinking	2.07	2.48	1.20
High alcohol risk drinking	2.19	2.70	1.23*
Hand washing when handling raw meat	2.04	2.46	1.21
Food insecurity in the last 12 months	1.94	2.50	1.29
Recommended daily fruit intake	2.06	2.45	1.19
Usual use of low fat, reduced fat or skim milk	2.03	2.41	1.19
Adequate physical activity	2.09	2.50	1.19
Use of public water as usual source of water	1.71	1.97	1.15
Current daily or occasional smoking	2.20	2.76	1.25*
Smoke free household	1.93	2.23	1.15
Recommended daily vegetable intake	1.80	2.00	1.11

**Health Status**			
Ever diagnosed with asthma	2.09	2.40	1.15
Current asthma	2.05	2.26	1.10
Overweight or obesity	2.05	2.43	1.18
Diabetes or high blood sugar	1.54	1.48	0.96
Self-rated health status as excellent, very good or good	1.88	2.20	1.17
High or very high psychological distress	1.96	2.60	1.32*

**Health Services**			
Emergency department care rated as excellent, very good or good.	2.36	2.77	1.17
Difficulties getting health care when needing it	1.64	1.86	1.13
Over night hospital care in the previous 12 months rated as excellent, very good or good.	2.04	2.75	1.35*

**Social Capital**			
Attended at least one community event in the previous six months	2.05	2.46	1.20
Trust most people	2.05	2.52	1.23*
Visit neighbours	2.13	2.51	1.18
Average design effects and ratio of average design effects	2.00	2.38	1.19

*top five design effect ratios

Source: New South Wales Health Survey Program, Centre for Epidemiology and Research, New South Wales Department of Health.

### Cost comparison

According to data from the New South Wales Population Health Survey in 2003, 14.9 diallings, 8.3 costed diallings, and 35.4 minutes of calling time are required to get one completed interview using LA-RDD sample, while 9.9 dialling, 6.6 costed diallings and 31.5 minutes of calling time are required to get one completed interview using EWP sample. To achieve the same precision as an assumed simple random sample of 1,000 respondents (an effective sample size is 1,000), on average twice the respondents are required for a LA-RDD sample (design effect = 2.0) and over two-and-one-third times more respondents are required for an EWP sample (design effect = 2.38).

In Table 4, costs were compared on the basis of the number of respondents required for LA-RDD and EWP samples per 1,000 effective sample size. On average, 892 more costed diallings occur in the LA-RDD sample; however, 69.5 hours less calling time are needed for the LA-RDD sample, leading to the LA-RDD sample costing A$1,212 (4.3%) less than the EWP sample per 1,000 effective sample. In the example using the high psychological distress indicator, which has a design effect ratio of 1.32, the LA-RDD sample is estimated to cost $4,350 (14.2%) less than the EWP per 1,000 effective sample.

**Table 4:** Comparison of number of dialling, calling time and cost for an assumed electronic white pages sample to achieve the same precision of estimates from an assumed effective sample size of 1,000 (equivalent of a simple random sample size 1,000)

	**Average sample (design effect ratio = 1.19)**		**High psychological distress (design effect ratio = 1.32)**	
	
	**LA-RDD n = 2,000**	**EWP n = 2,380**	**LA-RDD n = 1,960**	**EWP n = 2,600**
	
**Number of dialling**	29,800	23,562	29,204	25,740
Costed dialling *	16,600	15,708	16,268	17,160
**Calling time (in hours)**	1,180	1,249.5	1,156.4	1,365
**Cost†**	$26,920	$28,132	$26,382	$30,732

* excluding dialling of not connected numbers and dialling with no answer

† The costs is calculated as: A$0.20 × number of costed dialling + A$20 × number of calling hours.

Source: New South Wales Health Survey Program, Centre for Epidemiology and Research, New South Wales Department of Health.

## Discussion

### Sample representativeness

The distribution of age groups by gender for the unweighted LA-RDD sample is closer to the distributions for the population than the unweighted EWP sample in this study resulting in a smaller coefficient of variation in weights for the LA-RDD sample. After weighting, the demographic profile of the weighted LA-RDD sample is closer to the research population than the weighted EWP sample. The weighted EWP sample tends to cover the more socioeconomically advantaged respondents than the weighted LA-RDD sample. Therefore, LA-RDD sample could better retain the relationship among multiple variables, and potentially benefit both descriptive health estimates and analytical analysis in terms of reducing bias and improving precision of estimates.

### Differences in estimates

Even though post stratification weighting removes most of the potential bias for estimates from the EWP sample compared with the LA-RDD sample, difference in estimates still exist between the weighted EWP sample and weighted LA-RDD sample in this study. The differences between the weighted prevalence estimates from the EWP sample compared to the LA-RDD sample were up to 1.0% or more higher for three indicators (usual use of low or reduced fat or skim milk, trust most people, and visit neighbours in the past week) and 1.0% or more lower for two indicators (hand washing when handing raw meat, and current daily or occasional smoking) in the overall adult population.

Overall, there were larger design effects for the EWP sample than the LA-RDD sample across all but one of the 23 health indicators examined with the average design effect being 2.00 for LA-RDD sample and 2.38 for EWP sample (design effect ratio of 1.19).

### Cost comparison

Based on this study, it is estimated that with a design effect ratio of 1.19 up to 1.32, the LA-RDD sample would save up to 14.2% in costs, A$4,350 less than the EWP sample per 1,000 effective sample. Previous studies in Australia have either focused on the number of diallings or calling time [9,10]. Although the number of diallings is substantially more in LA-RDD sampling than EWP sampling, because of the increased number of out-of-scope numbers, this does not equate to increased dialling cost because many of these dials have no dialling cost (non-connected number and dialling of number without an answer). These previous studies also did not calculate design effect differences between RDD and EWP sampling or how they would affect the overall cost. The comparison of costs involved in costed calls and paid calling time for interviewers on the basis of achieving same precision level gives a clearer evaluation of cost-effectiveness in a LA-RDD survey and an EWP survey.

## Conclusion

This study shows that LA-RDD sampling has the advantage of obtaining a better representative sample of the population, leading to population health estimates with potentially reduced bias compared with EWP sampling, and would benefit other analyses involving multiple variables. On the basis of achieving the same precision for health estimates, LA-RDD sampling surveys costs less than EWP sampling surveys per 1,000 effective sample.

## List of abbreviations

RDD = random digital dialling; LA-RDD = list assisted random digital dialling; EWP = electronic white pages; CATI = computer assisted telephone interviewing.

## Competing interests

The author(s) declare that they have no competing interests.

## Authors' contributions

Baohui Yang and Margo Eyeson-Annan contributed equally to this work

## Pre-publication history

The pre-publication history for this paper can be accessed here:


